# Genetic association in female stress urinary incontinence based on proteomic findings: a case-control study

**DOI:** 10.1007/s00192-019-03878-0

**Published:** 2019-02-04

**Authors:** Theresa Reischer, Sukirthini Balendran-Braun, Sandra Liebmann-Reindl, Berthold Streubel, Wolfgang Umek, Heinz Koelbl, Marianne Koch

**Affiliations:** 1 Department of Obstetrics and Gynecology, Medical University of Vienna, Spitalgasse 23, 1090 Vienna, Austria; 2 Core Facility Genomics, Medical University of Vienna, Vienna, Austria; 3 Department of Pathology, Medical University of Vienna, Vienna, Austria; 4 Karl Landsteiner Institut fuer Spezielle Gynaekologie und Geburtshilfe, Vienna, Austria

**Keywords:** COL1A1, Genetic association, MMP1, SERPINA5, Serum proteome, Stress urinary incontinence, UMOD, Urinary proteome

## Abstract

**Introduction and hypothesis:**

Previous studies have indicated a hereditary component of stress urinary incontinence; however, evidence on candidate genes or single-nucleotide polymorphisms (SNPs) is scarce. We hypothesize a genetic association of female stress urinary incontinence based on significant differences of the urinary and serum proteomic pattern in the identical study population.

**Methods:**

Case-control study of 19 patients and 19 controls. We searched for known SNPs of SUI candidate genes (*COL1A1, MMP1, SERPINA5, UMOD*) in the database of short genetic variations and PubMed. Genomic DNA was isolated using QIAamp DNA Blood Midi Kit (Qiagen). We performed Sanger sequencing of selected exons and introns.

**Results:**

The rs885786 SNP of the *SERPINA5* gene was identified in 15 cases and 10 controls (*p* = 0.09). The rs6113 SNP of the *SERPINA5* gene was present in 4 controls compared to 0 cases (*p* = 0.105). The rs4293393, rs13333226 and rs13335818 SNPs of the *UMOD* gene were identified in five cases and two controls (*p* = 0.20), the rs1800012 SNP of the *COL1A1* gene in five cases versus four controls (*p* = 0.24) and the homozygous rs1799750 SNP of the *MMP1* gene in eight cases versus five controls (p = 0.18). The combination of the rs885786 SNP of the SERPINA5 gene and rs179970 SNP of the MMP1 gene was detected in ten cases versus five controls (*p* = 0.072).

**Conclusions:**

We found nonsignificant trends toward associations of SNPs on the *SERPINA5, UMOD* and *MMP1* gene and SUI.

**Electronic supplementary material:**

The online version of this article (10.1007/s00192-019-03878-0) contains supplementary material, which is available to authorized users.

## Introduction

Stress urinary incontinence (SUI) has an estimated prevalence of almost 50% in the female population aged 16–50 (range 12.5–79%). Prevalence rates then decline in older women, which is possibly related to an increase of mixed urinary incontinence symptoms [1–4]. Despite the high prevalence rate, the exact etiology of SUI remains unknown. Previous studies have indicated a possible hereditary component; however, evidence on potential candidate genes or single-nucleotide polymorphisms (SNPs) is still scarce [5–8]. According to a population-based cross-sectional study, daughters of mothers suffering from urinary incontinence had a relative risk of 1.5 for developing SUI [5], and heritability for SUI was estimated as 34–41% in twin studies (monozygotic and dizygotic) [6–8]. Strong genetic effects (estimated proportion of variance of susceptibility to lower urinary tract symptoms of 0.51; 95% CI 0.07–0.67) could be observed for urinary incontinence in a Swedish national population-based study of twins (monozygotic and dizygotic) including 42,582 participants; however, this study did not separately discuss SUI [7].

In addition, genetic association studies have aimed to identify gene polymorphisms associated with urinary incontinence. One systematic review and meta-analysis of genetic association studies could identify a significant association of the rs1800012 polymorphism of the *COL1A1* gene with SUI (OR 2.1) and prolapse (OR 1.3) [9]. Other genes, including *LAMC1, MMP1, MMP3* and *MMP9*, have been tested for association with urinary incontinence and prolapse [10–12]. Among these, only *MMP1* was described as being associated with stress urinary incontinence [13]. However, meta-analyses of polymorphisms of these genes have not shown significant effects, and the studies were furthermore reported as “prone to bias due to genotyping errors or population stratification” [9]. Other studies, which have not yet been replicated, reported significant associations between incontinence and the CAG copy number variant of the androgen receptor (*AR*) and the rs6313 SNP of *HTR2A* and between SUI and rs2165241 and rs1048661 variants of *LOX-L1* [14–16].

Before looking into a possible genetic association of SUI in our own patient population, we previously identified and published differences in their urine and serum proteome [17, 18].

We were able to identify six urinary proteins (encoded by the genes *SERPINA5, LRG1, GAA, UMOD, PPIA* and *KIAA0586*), which had a significantly different urinary abundance in SUI patients compared with controls (q-value < 0.25; logFC 1.11, logFC 3.91, logFC 1.24, logFC -4.87, logFC 1.96 and logFC -1.99, respectively) [18].

Serum analyses of the same patient population identified a total of 7012 different proteins over all serum samples. Of these, 33 proteins were found to be induced (meaning that they were detected in SUI samples, but not in controls), whereas 5 proteins were found to be depleted (meaning that they were detected in control samples, but not in SUI samples). Among others, *SERPINA5* protein was identified as being induced (detected in SUI, not in controls) [17].

Summarizing those previous findings, we found plasma serine protease inhibitor (encoded by the gene SERPINA5) in a significantly higher abundance in urine samples of SUI patients compared with controls, and we also found it induced in serum samples of the same patients. Plasma serine protease inhibitor is usually found in low abundance in urine and acts, among other functions, as a pro-inflammatory factor [19, 20]. We chose to investigate known SNPs of the underlying gene SERPINA5 because of the unusually high abundance in urine samples of SUI patients in our previous study and the fact that we also found it induced in serum samples of the same patients. The other protein which caught our attention was uromodulin (encoded by the gene UMOD), which is usually found in high abundance in urine, but which we found in significantly lower abundance in SUI patients compared with healthy controls. Uromodulin is involved in water and electrolyte balance and kidney innate immunity, and it is described as a preventive factor regarding urinary tract infections [19, 21]. We selected UMOD for genetic analysis because we found it in unusually low abundance in the urine of SUI patients. However, uromodulin was not found in different abundance in serum samples, as concentration levels in serum are generally negligible.

Findings from previous urine and serum proteomic analysis may suggest a possible inflammatory component of SUI; however, these results need to be replicated in larger populations before reaching any conclusions.

As SNPs on COL1A1 and MMP1 have been described as possibly being associated with SUI, we selected those SNPs in addition to known SNPs on UMOD and SERPINA5 [9].

The objective of our current study was to investigate a genetic component of SUI, based on our previous findings, which identified significant differences in the urine and serum proteome between patients with SUI and matched continent controls in the same population.

## Materials and methods

Ethical approval was obtained from the Ethics Committee of the Medical University of Vienna (1163/2017), and informed consent was obtained from all participants.

This case-control study is a follow-up study of two previously conducted and published studies comparing the urine and serum proteome of the same study population [17, 18]. Inclusion criteria were identical to the previous studies on the urinary and serum proteome. Demographic data were comparable; the small changes are due to two drop-outs because of missing blood samples. For SUI patients, inclusion criteria were: history of symptoms of SUI for at least 3 months (including a specific history of complaint of involuntary leakage on effort or exertion or on sneezing or coughing), positive provocation stress test (defined as an observed transurethral loss of urine simultaneous with a cough or Valsalva maneuver at a bladder volume of 300 ml), negative urine dipstick testing, age ≥ 18 years, patients capable of independent toileting, written informed consent and at least one previous vaginal delivery. Exclusion criteria were: previous treatment for SUI (surgical or pharmacological), history of overactive bladder symptoms and/or urinary incontinence other than SUI (tested using the ICIQ-short form questionnaire); neurological disorders potentially affecting the urinary tract system, such as multiple sclerosis or Parkinson’s disease; pelvic organ prolapse stage ≥ II (International Continence Society classification), clinically significant bladder outlet obstruction and/or post-void residual volume > 100 ml; history of acute urinary retention or history of repeated catheterizations, history of bladder cancer or previous surgery of the urinary tract; acute or recurrent urinary tract infection and/or hematuria; history of urinary tract stones; renal insufficiency and/or hepatic disease; history of alcohol and/or drug abuse; pregnancy or lactation; and any patient with a serious medical condition. The control group was formed by women without SUI or any other form of incontinence (ICIQ-short form score equal to zero and a negative cough stress test). Serum analyses for creatinine, transaminases and bilirubin values were undertaken, and only women with normal test results were included [18].

Blood samples were available from 19 patients with isolated SUI and age-matched controls (total *n* = 38). Samples were immediately centrifuged after collection to separate serum from blood cells and were subsequently frozen at −20 °C until further processing. A literature search was undertaken to identify candidate genes for SUI (*COL1A1, MMP1*) and their frequency of known SNPs [9]. Additionally, known SNPs for genes encoding proteins, which had previously shown a significantly different abundance in urine and serum of our own study population (*SERPINA5, UMOD*) [17, 18], were searched in the database of short genetic variations (dbSNP) and PubMed. Taking into account the small sample size, we chose relevant SNPs according to their minor allele frequency (MAF) of at least 10%. Genomic DNA was isolated from blood using QIAamp DNA Blood Midi Kit (Qiagen) according to the protocol. We performed Sanger sequencing of the selected exons and introns. All primers are listed in Supplement 1.

Statistical analysis was conducted using SPSS (version 21). For bivariate analyses between the two groups, the chi-square test was applied. If the minimal expected frequency was < 5, Fisher’s exact test was applied instead. Due to the observational and hypothesis-generating character of this study, we did not adjust for multiple testing [22]. The threshold for statistical significance was set at *p* < 0.05. The guidelines for genome-wide association studies (GWAS) suggest *p* < 5 × 10ˆ-8 for conventional GWAS [23]. Whereas in GWAS all SNPs of all genes are screened, we restricted the investigation to selected SNPs of single genes. This manuscript was structured according to the STROBE guidelines (for observational studies) [24].

## Results

Demographic data were similar to those of the previously published study comparing the serum proteome of patients with SUI and controls (Table 1) [17]. Cases and controls did not differ regarding age, BMI, parity, gravidity and vaginal delivery. There was no significant difference concerning the rate of chronic diseases or the number of postmenopausal patients in both groups.

**Table 1:** Demographic data

	SUI	Control	*p* value
	*n* = 19	*n* = 19	
(Mean ± SD)			
Age (years)	49.2 (± 9.6)	49.6 (± 9.7)	ns
BMI (kg/cm²)	27.6 (± 6.0)	24.8 (± 3.7)	ns
ICIQ* sum score	13.4 (± 3.9)	0 (0)	–
Gravidity	2.2 (± 0.9)	2.1 (± 0.8)	ns
Parity	2.2 (± 0.9)	2.0 (± 0.8)	ns
Vaginal deliveries (*n*)	2.1 (± 0.9)	1.8 (± 0.7)	ns
Chronic diseases (*n*)**	9/19 (47%)	11/19 (53%)	ns
Menopause			ns
Premenopausal	14/19 (74%)	12/19 (63%)	
Postmenopausal	5/19 (26%)	7/19 (37%)	

*ICIQ-UI Short Form (International Consultation on Incontinence Modular Questionnaire); **number of patients with chronic diseases (including hypertension, coronary heart disease, colitis, depression, gastritis, type II diabetes, glaucoma, chronic atrial fibrillation, asthma, Hashimoto thyroiditis, hyperthyroidism and factor V Leiden mutation)

**Fig. 1:**
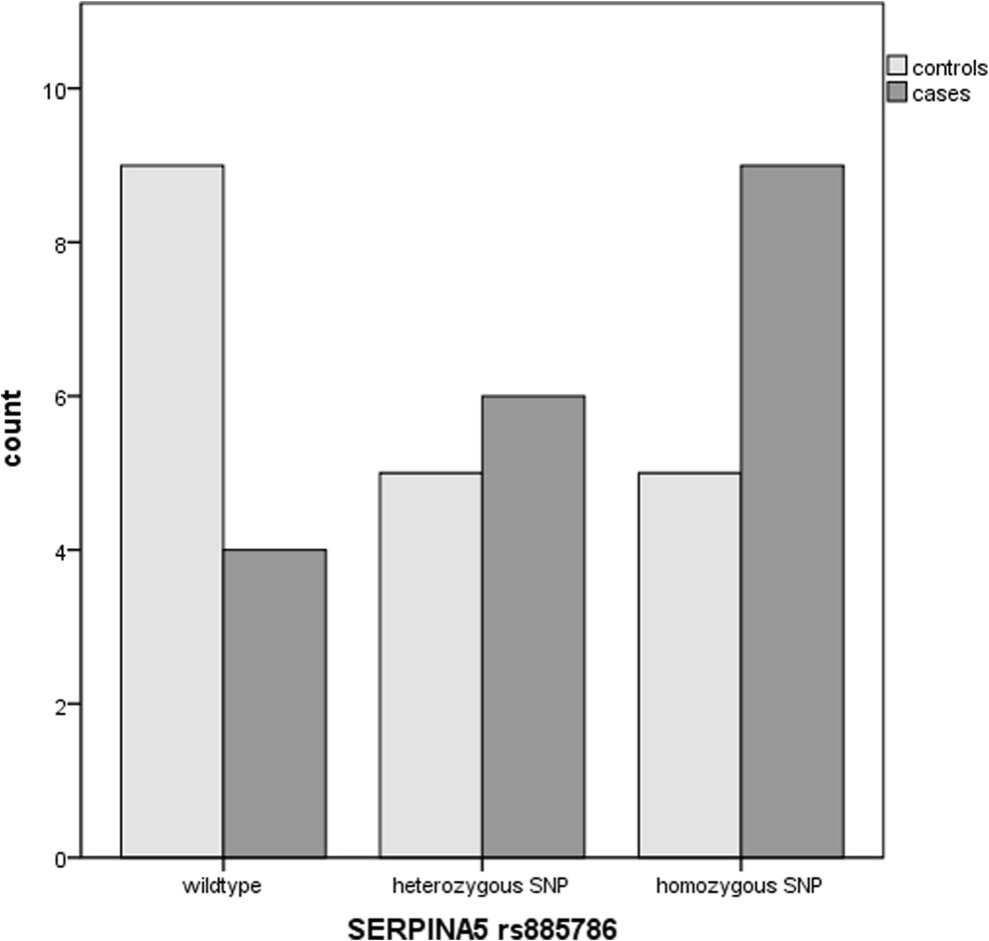
Identification of the rs885786 SNP of the *SERPINA5* gene in SUI patients and controls

The rs885786 (homozygous and heterozygous) SNP of the *SERPINA5* gene was identified in 15 cases and 10 controls (*p* = 0.09) (Fig. 1).

**Fig. 2:**
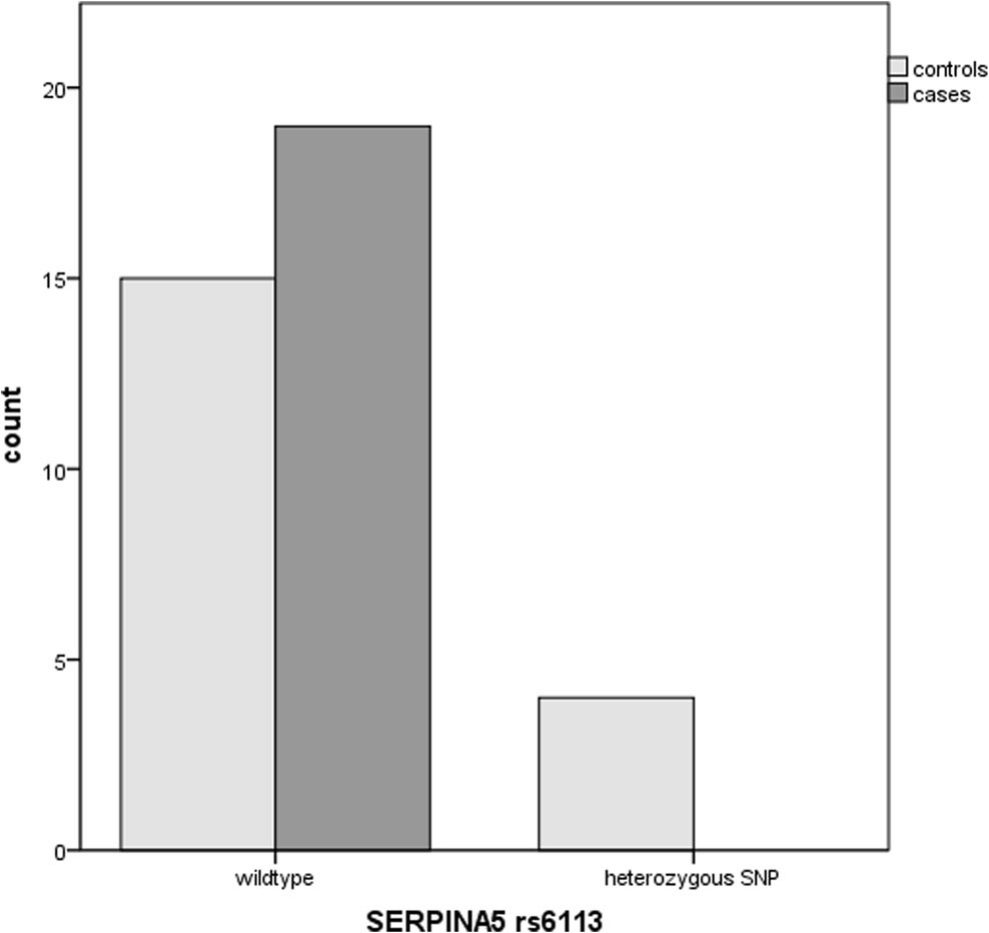
Identification of the rs6113 SNP of the *SERPINA5* gene in SUI patients and controls

The rs6113 SNP of the *SERPINA5* gene was present in four controls compared with no cases (*p* = 0.105) (Fig. 2).

**Fig. 3:**
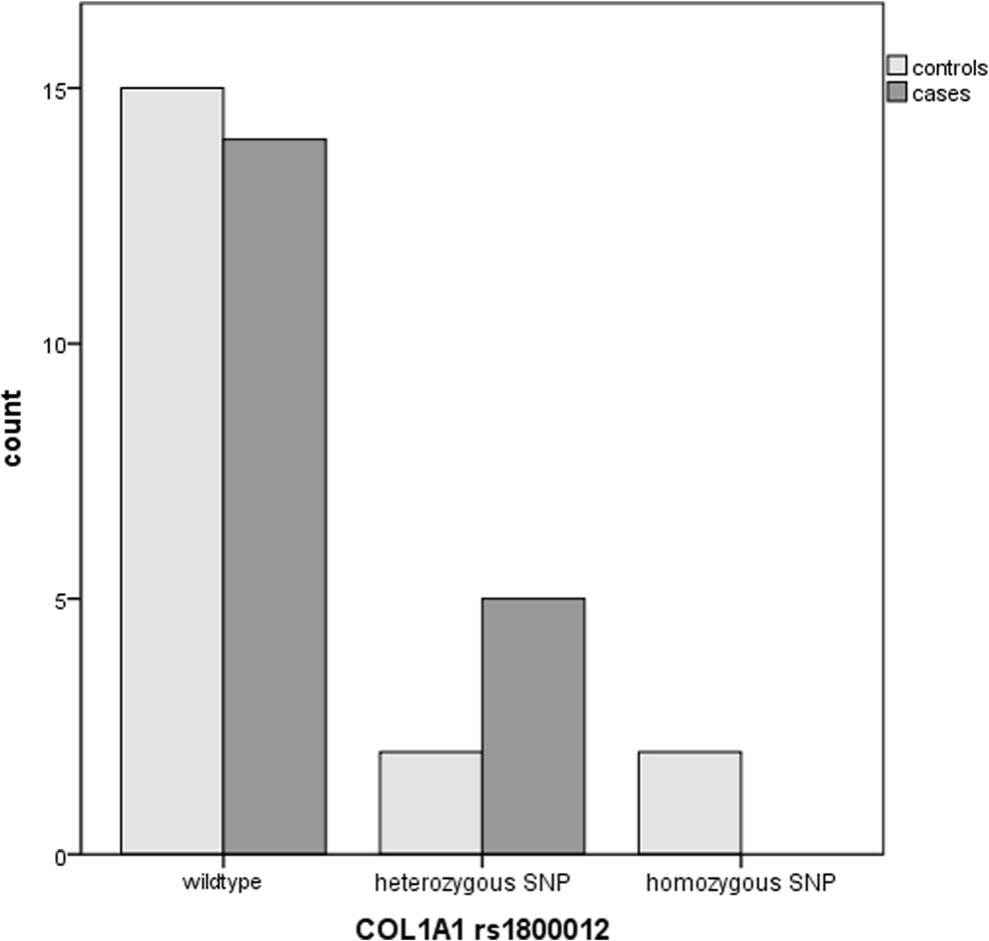
Identification of the rs1800012 SNP of the *COL1A1* gene in SUI patients and controls

Other known SNPs of the *SERPINA5* gene (rs10130906, rs2069963, rs2069962, rs2069961, rs2069959) did not show any trends in difference between the two groups as well as the rs11647727 and rs34857077 SNPs of the *UMOD* gene. The rs4293393, rs13333226 and rs13335818 (homozygous and heterozygous) of the *UMOD* gene were identified in five cases and two controls (*p* = 0.20). The rs1800012 SNP in the *COL1A1* gene was almost equally distributed and present in five cases versus four controls (heterozygous and homozygous) (*p* = 0.24) (Fig. 3).

**Fig. 4:**
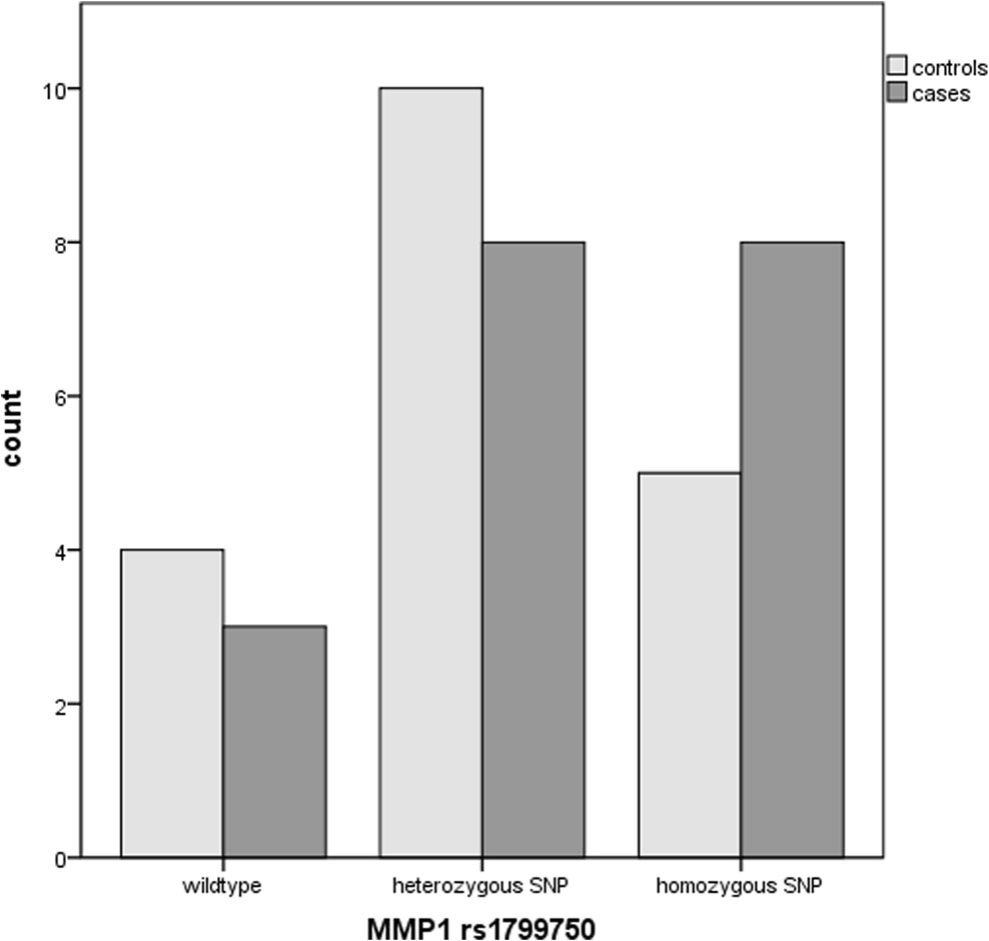
Identification of the rs1799750 SNP of the *MMP1* gene in SUI patients and controls

The homozygous rs1799750 SNP of the *MMP1* gene was present in eight cases versus five controls (*p* = 0.18) (Fig. 4).

**Fig. 5:**
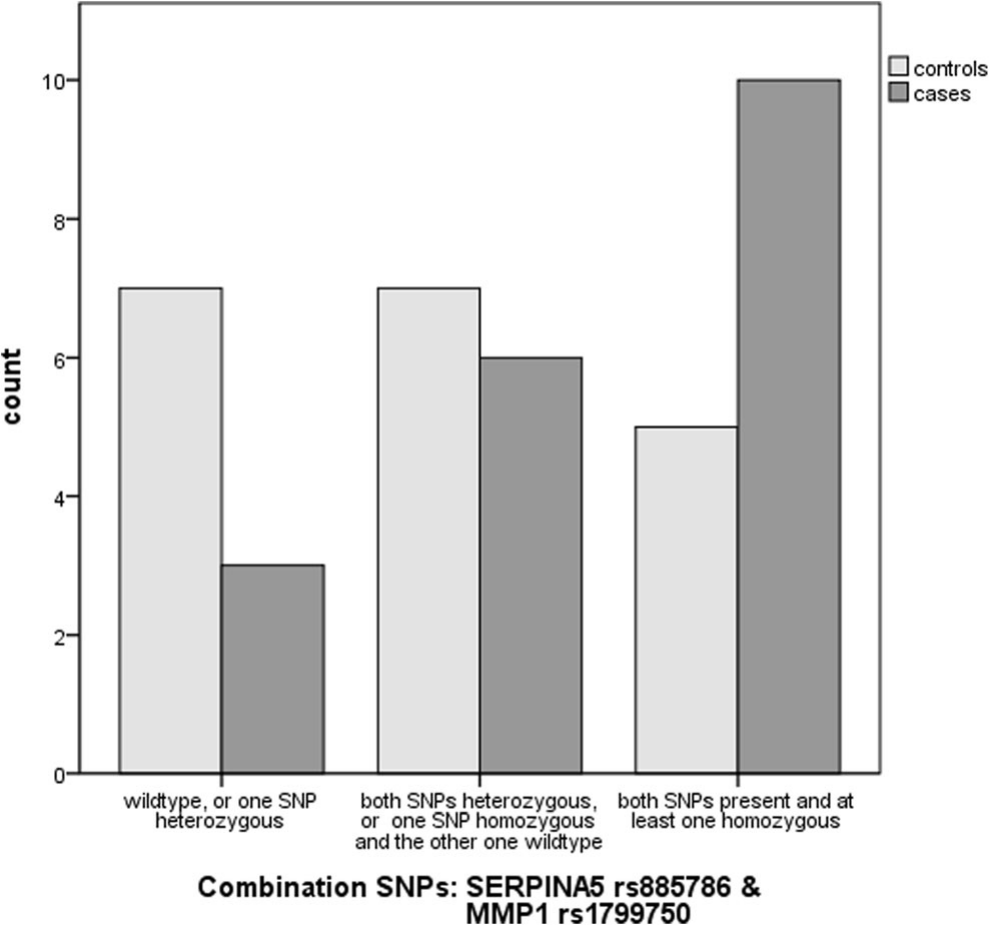
Identification of the combined presence of the rs885786 SNP of the *SERPINA5* gene and the rs179970 SNP of the *MMP1* gene

The combination of the rs885786 SNP of the SERPINA5 gene and rs179970 SNP of the MMP1 gene was detected in ten cases and five controls (*p* = 0.072). Overall we identified a non-significant trend toward an association of a combined presence of these SNPs and SUI (Fig. 5).

## Discussion

Previous studies have aimed to explore a genetic component of urinary incontinence by pre-selecting candidate genes according to their potential physiological or anatomical role in the dynamics of incontinence. In our study, however, we first identified and compared the urinary and serum proteome within the same population, and only according to these findings were candidate genes then selected.

Through proteomics, every protein present in a sample can be qualitatively and quantitatively identified, which allows an overview of the diseases’ pathophysiology, without the bias of pre-selection of potential candidate proteins. Following up on cumulative findings in urine and serum samples in the identical study population, we selected the genes *SERPINA5* and *UMOD* for genetic analysis. Several SNPs were identified in the past for both genes [25–30]. In addition, we aimed to replicate previous studies associating SNPs of *COL1A1* and *MMP1* genes to SUI [9, 31].

Results of this study did not show significant differences in SNPs of investigated genes when comparing SUI patients with healthy controls. We could only see non-significant trends toward an association of the rs885786 (homozygous and heterozygous) SNP of the *SERPINA5* gene and SUI as well as a non-significant trend toward an association of the combination of the rs885786 SNP of the SERPINA5 gene and rs179970 SNP of the MMP1 gene and SUI.

The*SERPINA5* gene encodes for the protein plasma serine protease inhibitor, which inactivates serine proteases and regulates intravascular as well as extravascular proteolytic activities. At this point, we can only speculate in which ways *SERPINA5* is potentially involved in the development of SUI, but it may support previous theories of inflammatory factors being involved.

The protein uromodulin encoded by the *UMOD* gene is usually highly abundant in urine and is known for its protective mechanisms such as prevention of urinary tract infections [21]. In our previous proteomic study, uromodulin was found in significantly lower abundance in urine of SUI patients [18]. A possible genetic association of the *UMOD* gene and SUI would therefore support the theory of a “lost protection” mechanism.

Strengths of this study include the strict selection of the study population. All SUI patients had isolated SUI (no urgency incontinence or pelvic organ prolapse), which had been tested by physical examination and the ICIQ-Urinary Incontinence Form [32]. Most genetic association studies, however, have included patients with any type of urinary incontinence and have not pre-selected the subtype of SUI. To our knowledge, no other study so far has reported results on a difference in the urinary and serum proteomic profile in SUI patients and has furthermore used this approach as the basis for a genetic association study.

One limitation of our study is the relatively small sample size, which was adequately calculated for the proteomics analysis, but which is too small to allow conclusions on genetic associations. Even though we could identify trends, we were not able to show any significant associations.

SNPs on the SERPINA5 gene may support a previously established theory of inflammatory processes leading to SUI, whereas SNPs on the UMOD gene may support the theory of a “lost protection” mechanism.

So far, we have identified a different urinary and serum proteome in patients with SUI compared with controls. However, it is impossible to say whether the differing proteome has a causal relationship to the disease or is a result of genetic variants. Thus, larger studies with bigger sample sizes investigating possible associations of SNPs on these specific genes and SUI are needed.

## Electronic supplementary material

Supplement 1 List of primer (XLSX 14 kb)

## References

[1] Abrams P, Cardozo L, Wein A (2014). Fourth international consultation on Incontinence-Research Society 2013. Neurourol Urodyn.

[2] Haylen BT, de Ridder D, Freeman RM, Swift SE, Berghmans B, Lee J, Monga A, Petri E, Rizk DE, Sand PK, Schaer GN (2010). An International Urogynecological Association (IUGA)/International Continence Society (ICS) joint report on the terminology for female pelvic floor dysfunction. Int Urogynecol J.

[3] Garely AD, Noor N (2014). Diagnosis and surgical treatment of stress urinary incontinence. Obstet Gynecol.

[4] Almousa S, Bandin van Loon A (2018). The prevalence of urinary incontinence in nulliparous adolescent and middle-aged women and the associated risk factors: a systematic review. Maturitas.

[5] Hannestad YS, Lie RT, Rortveit G, Hunskaar S (2004). Familial risk of urinary incontinence in women: population based cross sectional study. Bmj.

[6] Altman D, Forsman M, Falconer C, Lichtenstein P (2008). Genetic influence on stress urinary incontinence and pelvic organ prolapse. Eur Urol.

[7] Wennberg AL, Altman D, Lundholm C, Klint A, Iliadou A, Peeker R, Fall M, Pedersen NL, Milsom I (2011). Genetic influences are important for most but not all lower urinary tract symptoms: a population-based survey in a cohort of adult Swedish twins. Eur Urol.

[8] Rohr G, Kragstrup J, Gaist D, Christensen K (2004). Genetic and environmental influences on urinary incontinence: a Danish population-based twin study of middle-aged and elderly women. Acta Obstet Gynecol Scand.

[9] Cartwright R, Kirby AC, Tikkinen KA, Mangera A, Thiagamoorthy G, Rajan P, Pesonen J, Ambrose C, Gonzalez-Maffe J, Bennett P, Palmer T, Walley A, Jarvelin MR, Chapple C, Khullar V (2015). Systematic review and metaanalysis of genetic association studies of urinary symptoms and prolapse in women. Am J Obstet Gynecol.

[10] Ferrari MM, Rossi G, Biondi ML, Vigano P, Dell’utri C, Meschia M (2012). Type I collagen and matrix metalloproteinase 1, 3 and 9 gene polymorphisms in the predisposition to pelvic organ prolapse. Arch Gynecol Obstet.

[11] Nikolova G, Lee H, Berkovitz S, Nelson S, Sinsheimer J, Vilain E, Rodriguez LV (2007). Sequence variant in the laminin gamma1 (LAMC1) gene associated with familial pelvic organ prolapse. Hum Genet.

[12] Skorupski P, Jankiewicz K, Miotla P, Marczak M, Kulik-Rechberger B, Rechberger T (2013). The polymorphisms of the MMP-1 and the MMP-3 genes and the risk of pelvic organ prolapse. Int Urogynecol J.

[13] Vishwajit S, Rohozinski J, Andersson K-E, Badlani GH (2009). Association of MMP1 promoter variant with stress urinary incontinence and pelvic organ prolapse in women. J Urol.

[14] Cornu JN, Merlet B, Cussenot O, Cancel-Tassin G, Ciofu C, Amarenco G, Haab F (2011). Genetic susceptibility to urinary incontinence: implication of polymorphisms of androgen and oestrogen pathways. World J Urol.

[15] Noronha JA, Schwanke CH, Machado DC, Braga R, Lubianca JM, Sesti FL, de Toledo AF, da Cruz IB (2010). Association between T102C polymorphism of serotonin 2A receptor gene and urinary incontinence in older women. J Investig Med Off Publ Am Fed Clin Res.

[16] Ozbek E, Polat EC, Ozcan L, Otunctemur A, Emrence Z, Ustek D (2013). TT polymorphism in rs2165241 and rs1048661 region in lysyl oxidase like-1 gene may have a role in stress urinary incontinence physiopathology. J Obstet Gynaecol Res.

[17] Koch Marianne, Umek Wolfgang, Hanzal Engelbert, Mohr Thomas, Seyfert Sonja, Koelbl Heinz, Mitulović Goran (2018). Serum proteomic pattern in female stress urinary incontinence. ELECTROPHORESIS.

[18] Koch M, Mitulovic G, Hanzal E, Umek W, Seyfert S, Mohr T, Koelbl H, Laterza RM (2016). Urinary proteomic pattern in female stress urinary incontinence: a pilot study. Int Urogynecol J.

[19] The UniProt C (2017). UniProt: the universal protein knowledgebase. Nucleic Acids Res.

[20] Laurell M, Christensson A, Abrahamsson PA, Stenflo J, Lilja H (1992). Protein C inhibitor in human body fluids. Seminal plasma is rich in inhibitor antigen deriving from cells throughout the male reproductive system. J Clin Invest.

[21] Scolari F, Izzi C, Ghiggeri GM (2015). Uromodulin: from monogenic to multifactorial diseases. Nephrol Dial Transplant Off Publ Eur Dial Transplant Assoc Eur Ren Assoc.

[22] Bender R, Lange S (2001). Adjusting for multiple testing—when and how?. J Clin Epidemiol.

[23] Barsh GS, Copenhaver GP, Gibson G, Williams SM (2012). Guidelines for genome-wide association studies. PLoS Genet.

[24] von Elm E, Altman DG, Egger M, Pocock SJ, Gotzsche PC, Vandenbroucke JP, Initiative S (2008). The strengthening the reporting of observational studies in epidemiology (STROBE) statement: guidelines for reporting observational studies. J Clin Epidemiol.

[25] Pattaro C, De Grandi A, Vitart V, Hayward C, Franke A, Aulchenko YS, Johansson A, Wild SH, Melville SA, Isaacs A, Polasek O, Ellinghaus D, Kolcic I, Nothlings U, Zgaga L, Zemunik T, Gnewuch C, Schreiber S, Campbell S, Hastie N, Boban M, Meitinger T, Oostra BA, Riegler P, Minelli C, Wright AF, Campbell H, van Duijn CM, Gyllensten U, Wilson JF, Krawczak M, Rudan I, Pramstaller PP, consortium E (2010). A meta-analysis of genome-wide data from five European isolates reveals an association of COL22A1, SYT1, and GABRR2 with serum creatinine level. BMC Med Genet.

[26] Cui L, Bai Y, Xu J, Zhang J, Zhang H, Zhang S, Zhang W (2015). Single-nucleotide polymorphism of the UMOD promoter is associated with the outcome of chronic kidney disease patients. Biomed Rep.

[27] Kim S, Swaminathan S, Inlow M, Risacher SL, Nho K, Shen L, Foroud TM, Petersen RC, Aisen PS, Soares H, Toledo JB, Shaw LM, Trojanowski JQ, Weiner MW, McDonald BC, Farlow MR, Ghetti B, Saykin AJ, Alzheimer’s Disease Neuroimaging I (2013). Influence of genetic variation on plasma protein levels in older adults using a multi-analyte panel. PLoS One.

[28] Kottgen A, Yang Q, Shimmin LC, Tin A, Schaeffer C, Coresh J, Liu X, Rampoldi L, Hwang SJ, Boerwinkle E, Hixson JE, Kao WH, Fox CS (2012). Association of estimated glomerular filtration rate and urinary uromodulin concentrations with rare variants identified by UMOD gene region sequencing. PLoS One.

[29] Sigurdson AJ, Brenner AV, Roach JA, Goudeva L, Muller JA, Nerlich K, Reiners C, Schwab R, Pfeiffer L, Waldenberger M, Braganza M, Xu L, Sturgis EM, Yeager M, Chanock SJ, Pfeiffer RM, Abend M, Port M (2016). Selected single-nucleotide polymorphisms in FOXE1, SERPINA5, FTO, EVPL, TICAM1 and SCARB1 are associated with papillary and follicular thyroid cancer risk: replication study in a German population. Carcinogenesis.

[30] Vilander LM, Kaunisto MA, Vaara ST, Pettila V, group Fs (2017). Genetic variants in SERPINA4 and SERPINA5, but not BCL2 and SIK3 are associated with acute kidney injury in critically ill patients with septic shock. Crit Care.

[31] Skorupski P, Krol J, Starega J, Adamiak A, Jankiewicz K, Rechberger T (2006). An alpha-1 chain of type I collagen Sp1-binding site polymorphism in women suffering from stress urinary incontinence. Am J Obstet Gynecol.

[32] Avery K, Donovan J, Peters TJ, Shaw C, Gotoh M, Abrams P (2004). ICIQ: a brief and robust measure for evaluating the symptoms and impact of urinary incontinence. Neurourol Urodyn.

